# Rationale and design of individualized quality improvement based on the Computer Analysing system to improve Stroke management quality Evaluation (CASE): a multicenter historically controlled study

**DOI:** 10.1186/s13063-020-04598-3

**Published:** 2020-07-24

**Authors:** Yi Chen, Wansi Zhong, Xiaoxian Gong, Haitao Hu, Shenqiang Yan, Xuting Zhang, Zhicai Chen, Ying Zhou, Min Lou

**Affiliations:** Department of Neurology, The Second Affiliated Hospital of Zhejiang University School of Medicine, 88# Jiefang Road, Hangzhou, 310009 China

**Keywords:** Acute ischemic stroke, Computer-based analyzing system, Key performance indicator, Medical quality improvement

## Abstract

**Background:**

Guideline-based medical care has been identified to improve outcomes in stroke. However, data acquisition and medical quality management during hospital stay still need to be improved in China. We have developed a computer-based medical data collecting system, together with automated calculation of key performance indicators (KPIs) and regular individualized education, and thus aim to explore whether it can improve the medical care quality of acute ischemic stroke (AIS) during hospital stay in stroke centers.

**Methods:**

The individualized quality improvement based on the Computer Analysing system to improve Stroke management quality Evaluation (CASE) trial is a prospective, multicenter, historical control study among 30 stroke centers in China. In this trial, the data is directly extracted from the saved original medical record of each AIS patient during hospital stay, regardless of different Electronic Medical Record System (EMRS) in each center. Then, the automated calculation of KPIs and the regular education via teleconference per month allow the clinicians to examine the causes of non-compliance of guideline-based care and develop programs to decrease their frequency.

**Discussion:**

We compare KPIs between pre-intervention stage and post-intervention stage (without or with education) among stroke centers. If proved effective, this approach might be generalized around China and even worldwide, where a unified EMRS is difficult to be applied and in-patient care needs to be improved.

**Trial registration:**

ClinicalTrials.gov NCT03684629. Registered on 9 December 2018. Retrospectively registered.

## Background

Stroke burden has increased over the past three decades in China, emerging to be the leading cause of mortality [1, 2]. A systematic review indicated that implementing clinical guidelines improved the quality and efficiency of medical care [3]. Moreover, studies such as GOLDEN BRIDGE [4], The Get With The Guidelines (GWTG) [5], and BRIDGE STROKE [6] also indicated that increased delivery of in-patient care based on guidelines could contribute to the favorable outcome of stroke patients [7].

To enhance the medical care quality by increasing the compliance with evidence-based performance in stroke patients, China Stroke Center Alliance (CSCA) has been launched in China in 2015. CSCA prompted 13 key performance indicators (KPIs) of acute ischemic stroke (AIS) to monitor the routine in-patient medical care quality, based on the AIS guidelines, involving intravenous thrombosis (IVT), antiplatelet therapy, anticoagulant therapy, and so on [8–13]. Then afterwards, CSCA annually collected the required data from about 20,000 in-patients among more than 1500 stroke centers [14], and calculated and feedback all of the KPIs to each center.

However, there is a huge diversity in the Electronic Medical Record System (EMRS) in different stroke centers in China currently, which makes it difficult to capture all KPIs from the original medical records by a unified system in each center. Accordingly, clinicians in each stroke center have to report the medical data online manually to achieve the feedback of KPIs from CSCA. Indeed, this is also the way data was collected in the above studies (GOLDEN BRIDGE, GWTG, BRIDGE STROKE).

Obviously, this kind of collection has its inherent shortcomings, including the vast cost of manpower and time from clinicians, and questioning of its authenticity due to the lack of traceability of original medical records and enough quality control of data. To minimize the above limitations, we thus developed a computer-based medical data collecting system, with subsequent automated calculation of KPIs and regular individualized education to improve the adherence to guideline-based care. The main purpose is to decrease the workload of clinicians in stroke centers on reporting data manually. We designed this individualized quality improvement based on the Computer Analysing system to improve Stroke management quality Evaluation (CASE) trial to explore whether this novel approach can improve inpatient medical care quality of AIS in each stroke center.

## Method and design

### Design

This is an 18-month prospective, multicenter, historical control study among 30 stroke centers in Zhejiang Province, China. Stroke centers with in-hospital stroke patients voluntarily participate in this trial. All medical documents of consecutive AIS patients admitted in the stroke center according to ICD-10 (I63 and G45) are collected by investigators from Zhejiang Stroke Quality Control Center (ZSQCC). Only the de-identified documents are preserved in a safe information database. The clinical data involved in this study belong to the scope of information collection of routine clinical practice. The local infrastructure and characteristics of each recruited center are also recorded. The study protocol is approved by the Institutional Review Board of the Second Affiliated Hospital of Zhejiang University (SAHZU). Because patient information in the CASE is de-identified and anonymized before being released to the researchers, the informed consent requirement is waived by SAHZU Institutional Review Board. All the protocol item is standardized (see Additional file 1). It was registered at ClinicalTrials.gov (NCT03684629).

**Fig. 1:**
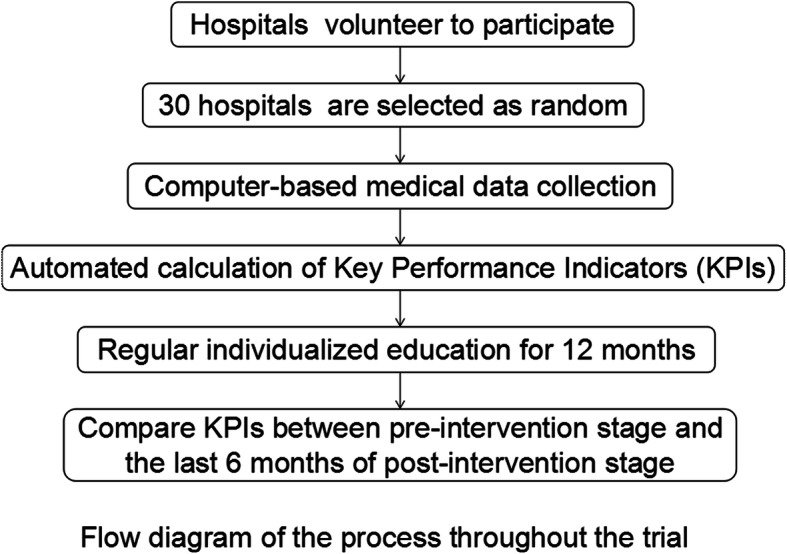
Flow diagram of the process throughout the trial

There are two stages in this trial: pre-intervention stage (the initial 6 months) and post-intervention stage (the next 12 months). We collect the consecutive data of each center during the pre-intervention stage without feedback of KPIs and then monthly send its own KPIs to each center during the post-intervention stage. We finally compare the changes of KPIs from pre-intervention stage to the last 6 months of post-intervention stage (Fig. 1).

### The inclusion criteria for the analysis of medical documents

1. Patients 18 years or older
2. AIS confirmed by computed tomography (CT) or diffusion-weighted imaging (DWI)
3. Admitted within 7 days from symptom onset

### Data monitoring and confidentiality

**Fig. 2:**
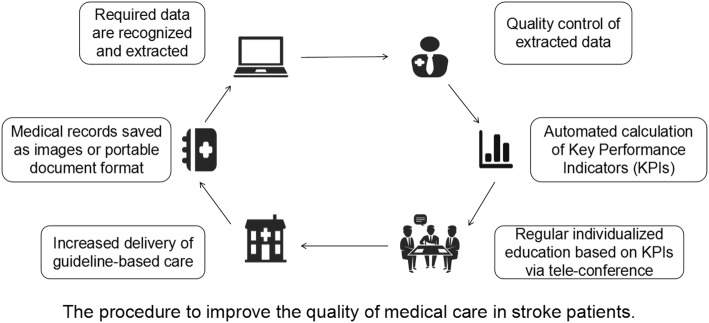
The procedure to improve the quality of the medical care in stroke patients

After discharge, the original medical document of each patient during hospital stay is saved as images or portable document format (PDF). Specific software recognizes and pre-processes the above material and sends it to multiple Optical Character Recognition (OCR) [15] engines to build documents with recognized text, which are subsequently re-segmented and synthesized in the post-processing. Required data is extracted from the processed text. Three hundred medical documents were randomly selected to evaluate the error rate of the extraction, i.e., the rate of inconsistent data extracted by computer-based medical data collecting system and manual input of neurologists, which was finally 3.4%. Previous report has also demonstrated generalizability and applicability of OCR with a positive predictive value of 94.6% [16]. This software also allows the trained study investigators to quickly check the quality of extracted data by pointing out the original extraction site, enabling the authenticity and traceability of original data. The checked data are then imported into the analyzing system, and KPIs will be calculated according to the designed formula (Fig. 2).

### Intervention for medical care improvement

Individualized quality improvement process is divided into 3 steps:

1. Each stroke center receives their own KPIs monthly, as well as the average KPI level of all 30 stroke centers as contrast, which are automatically calculated by computer analyzing system.
2. Based on the performance of KPI data, stroke experts from ZSQCC identify the items which need to be improved, provide each center with relevant education of guidelines, and help the clinicians to examine the causes of non-compliance of guideline-based care and develop programs to decrease their frequency via regular teleconference per month and face-to-face lectures quarterly. In the meantime, stroke centers with the best KPI performance also share their experiences on medical care improvement.
3. Each stroke center is then required to provide a detailed improvement plan for each KPI and check whether it is implemented one by one during the next teleconference.

### Outcome

The primary outcomes are as follows: 7 KPIs including the accomplishment of National Institutes of Health Stroke Scale (NIHSS), intravenous thrombosis within 4.5 h of onset, antiplatelet agent therapy within the first 48 h, statin use after hospitalization and at discharge, antithrombotic therapy at discharge, and anticoagulant treatment for patients with atrial fibrillation at discharge [8, 9, 11–13] (see Additional file 2).

The secondary outcomes are as follows: 9 KPIs including prevention of deep vein thrombosis, blood vessel evaluation within 1 week of hospitalization, swallowing function evaluation, blood lipid level assessment, rehabilitation evaluation and implementation, recommendation of life style change, anti-hypertensive therapy, hypoglycemic treatment at discharge, and recurrence rate [10, 11, 17] (see Additional file 2).

### Sample size

Among all the KPI, the adherence to intravenous thrombosis (IVT) was poor in china [18]. Result of investigation in Zhejiang before the trial indicated that only approximately 8% of ischemic stroke patients received IVT 4.5 h after stroke onset. To detect 10% improvement in the adherence to IVT, with *α* = 0.05 and power = 90%, at least 16,570 patients will be needed in pre-intervention stage and post-intervention stage.

### Planned data statistical analysis

**Fig. 3:**
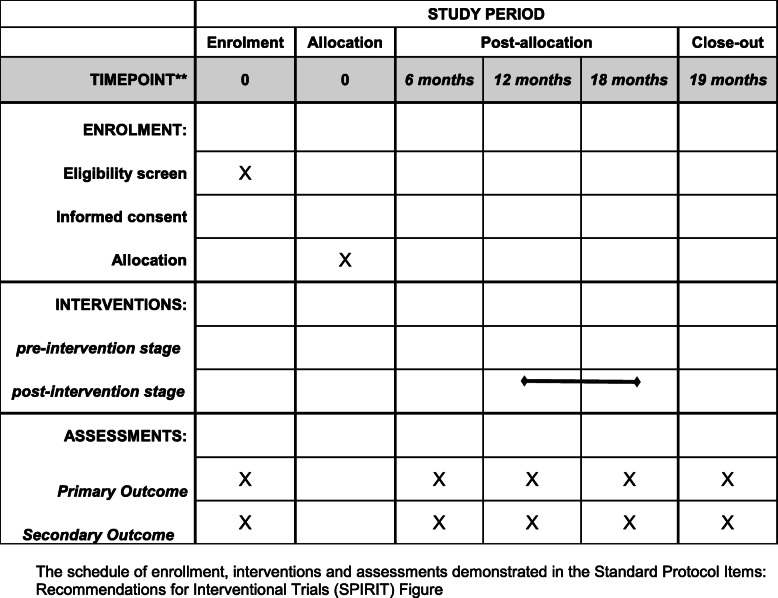
The schedule of enrolment, interventions, and assessments

Statistical analysis will be performed with SPSS 22.0 (SPSS, Inc., Chicago, USA). Baseline characteristics of each stroke center (see Additional file 3) and the patients enrolled will be analyzed. The achieved rates of all KPIs of 30 hospitals during pre-intervention stage and the last 6 months of post-intervention stage are compared by the paired *t* test. KPIs are also collected at multiple time points before and after intervention. Interrupted time series (ITS) analysis is performed to assess the efficiency on outcome 1 year after the intervention is carried out [19, 20]. The multiple time points before the intervention allow the underlying trend to be estimated, and the multiple time points after the intervention allow the intervention effect to be estimated accounting for the underlying trend. No additional adjusted analyses will be performed. All the information to calculate the KPIs was included in the medical records. The missing data not extracted by a computer will be extracted manually by a clinician. There are no anticipated problems that are detrimental to the participant so that there are no formal stopping rules for the trial. The schedule of enrolment, interventions, and assessments is included in Fig. 3.

### Trial management and monitoring

The Trial Management Group (TMG) will be responsible for all aspects of local organization and the day-to-day running of the trial, including monitoring the operation and maintenance of the computer analyzing system, outcome data collection, and ensuring the feedback of KPIs to the relevant hospitals every month. The trial will be overseen by an independent Trial Steering Committee (TSC), which will meet at least monthly to consider and address strategic issues. A Data Monitoring Committee (DMC), members of which will act independently of the TSC, TMG, and funder, will monitor data, check the quality of extracted data, and make recommendations to the TSC on whether there are any ethical or safety reasons why the trial should not continue. The serious adverse events (SAEs) which will be recorded and reported in this trial are deaths by any cause in the 1 year after hospitalization. The DMC will monitor the occurrence of SAEs. Patient information in the CASE is de-identified and anonymized, extracted from medical document after discharge.

## Discussion

It is widely agreed that accuracy and reliability are of importance for data collection, which may interfere with the sample size and data quality for research [21]. Manual report can lead to data errors, and the authenticity of data cannot be guaranteed without verification of original medical record. Even the Get With The Guidelines (GWTG)-Stroke program, a national stroke quality improvement for AIS and TIA via the web, also has missing data during the hospital participation in the program, possibly due to the manual input [22]. Importantly, it is difficult for the clinicians to insist on quality improvement based on their manual reporting after busy clinical practice, when faced with a huge number of patients. Currently, it is also difficult to deliver automated calculation of KPIs via a unified EMRS around China. In CASE trial, data are directly extracted from the saved original record of each patient based on optimized OCR, having nothing to do with the work of EMRS itself. Theoretically, this approach can be implemented in all centers and the extraction saves the time of all clinicians.

Based on the reliable feedback, regular individualized education would further improve the adherence to guideline-based care [23, 24]. By providing the participating hospitals with improvement consultation, workshops, and webinars based on guidelines, GWTG-Stroke program achieved significant improvement of composite measure of care from 83.53 to 93.97% [25]. Data analysis from 2010 to 2013 in Germany showed that the mortality was reduced by up to 6.26 (63.7%) deaths per year, if the average hospital boosts its performance to an efficient treatment of stroke patients [26]. In CASE trial, the automated calculation of KPIs identifies the non-compliance of guideline-based care, which allows the clinicians to examine their cause and develop programs to decrease their frequency. The teleconference per month and the requirement of detailed improvement plan from each center would constitute into an upgraded dynamic feedback and improving system, leading to the enhanced surveillance and monitoring, and decreased medical care errors.

Therefore, it is reasonable to assume that this novel computer-based medical data collecting system, together with subsequent automated calculation of KPIs and regular individualized education, could increase the adherence to guideline-based care in each stroke center, finally improving the efficiency and quality of medical care of stroke patients.

This study has some limitations. First, it is a historical control study; thus, the quality of care in individual hospital may have changed over time, which can influence the general results of the trial. Second, the 30 hospitals were selected randomly from 78 voluntary hospitals rather than all hospitals in Zhejiang province. Randomizing has prevented issues around selection bias.

## Conclusion

In summary, this is a new mode to improve in-patient medical care, which consists of advanced acquisition technique and personalized feedback with persistent education. It can yield real benefits in terms of increased delivery of guideline-based care and reduced manual reporting of clinicians. If proved effective in Zhejiang province, this new quality improvement approach might be generalized around China and even worldwide, where a unified EMRS is difficult to be applied.

### Trial status

The protocol version number is 1.0, finalized in July 2018. The date of recruitment began on August 16, 2018. The approximate date when recruitment will be completed is January 2020.

## Supplementary information

**Additional file 1.** SPIRIT 2013 Checklist: Recommended items to address in a clinical trial protocol and related documents.

**Additional file 2.** The primary and secondary outcome measures.

**Additional file 3.** The baseline characteristics of primary stroke center (PSC) and comprehensive stroke center (CSC).

## Data Availability

The dataset is not publicly available due to confidentiality policies.

## Abbreviations

KPI: Key performance indicator
AIS: Acute ischemic stroke
CASE: The individualized quality improvement based on the Computer Analysing system to improve Stroke management quality Evaluation
EMRS: Electronic Medical Record System
GWTG: The Get With The Guidelines
CSCA: China Stroke Center Alliance
IVT: Intravenous thrombosis
ZSQCC: Zhejiang Stroke Quality Control Center
SAHZU: The Second Affiliated Hospital of Zhejiang University
CT: Computed tomography
DWI: Diffusion-weighted imaging
PDF: Portable document format
OCR: Optical Character Recognition
NIHSS: National Institutes of Health Stroke Scale
ITS: Interrupted time series
