# Supplementary material for: Rationale and design of individualized quality improvement based on the Computer Analysing system to improve Stroke management quality Evaluation (CASE): a multicenter historically controlled study
Source: Trials. 2020 Jul 24;21:677. doi: 10.1186/s13063-020-04598-3 (PMC7379356; doi:10.1186/s13063-020-04598-3)
Supplement: Supplementary file 2 — Additional file 2. The primary and secondary outcome measures. [file 13063_2020_4598_MOESM2_ESM.docx]

**Additional file 2: The primary and secondary outcome measures**

**Primary Outcome Measures:**

1. Completion rate of National Institutes of Health Stroke Scale (NIHSS)

Completion rate of NIHSS= ($\sum$AIS patients who completed assessment of NIHSS /$\sum$the total number of cerebral infarction patients hospitalize in the same period)*100%

1. Rate of intravenous recombinant tissue plasminogen activator (rt-pa) within 4.5 hours of onset

Rate of intravenous tPA within 4.5 hours of onset= ($\sum$AIS patients who received intravenous rt-pa thrombolytic therapy within 4.5 hours/$\sum$cerebral infarction who were admitted to hospital within 4.5 hours in the same period)*100%

1. Rate of administration of aspirin or other antiplatelet agents within 48 hours of admission

Rate of administration of aspirin or other antiplatelet agents within 48 hours of admission= ($\sum$AIS patients who received aspirin or other antiplatelet agents within 48 hours of admission/$\sum$the total number of cerebral infarction patients hospitalized in the same period)*100%

1. Rate of statin use during hospitalization

Rate of statin use during hospitalization= ($\sum$patients hospitalized for cerebral infarction who were treated with statins /$\sum$the total number of cerebral infarction patients hospitalized in the same period)*100%

1. Rate of statin treatment at discharge

Rate of statin treatment at discharge= ($\sum$patients with cerebral infarction who received statin at discharge/$\sum$the total number of patients with non-cardiac infarction who were hospitalized in the same period)*100%

1. Rate of antithrombotic therapy at discharge

Rate of antithrombotic therapy at discharge= ($\sum$patients with cerebral infarction who received antithrombotic drugs (such as aspirin, other antiplatelet agents, heparin, warfarin or new oral anticoagulants) at discharge/$\sum$the total number of hospitalized patients with cerebral infarction in the same period.)*100%

1. The rate of anticoagulant treatment for patients with atrial fibrillation at discharge

The rate of anticoagulant treatment for patients with atrial fibrillation at discharge= ($\sum$patients with cerebral infarction complicated with atrial fibrillation who received anticoagulants (e.g., heparin, low molecular heparin, warfarin, new oral anticoagulants) at discharge/$\sum$the total number of patients with cerebral infarction complicated with atrial fibrillation treated in hospital in the same period)*100%

**Secondary Outcome Measures:**

1. Rate of prevention of deep vein thrombosis

Rate of prevention of deep vein thrombosis= ($\sum$ patients who were unable to get out of bed for cerebral infarction within 48 hours of admission that were given prophylactic measures including heparin and/or DVT (Deep vein thrombosis) /$\sum$patients who were unable to get out of bed for cerebral infarction treated in hospital in the same period)*100%

1. Rate of blood vessel assessment within one week of hospitalization

Rate of blood vessel assessment within one week of hospitalization= ($\sum$patients with cerebral infarction hospitalized for 1 week who had completed the evaluation of the blood vessels in the neck and cranial (such as the ultrasound of the blood vessels in the neck or cranial, CT or MR angiography, or DSA)/$\sum$the total number of cerebral infarction patients hospitalized in the same period)*100%

1. Rate of swallowing function evaluation

Rate of swallowing function evaluation

= ($\sum$patients who had a history of drinking water test /$\sum$the total number of stroke patients who were hospitalized in the same period)*100%

1. Rate of lipid level assessment

Rate of lipid level assessment= ($\sum$patients who were assessed on lipid level during hospitalization /$\sum$the total number of patients who were hospitalized during the same period)*100%

1. Rate of rehabilitation evaluation and implementation

Rate of rehabilitation evaluation and implementation= ($\sum$patients who received rehabilitation evaluation and implementation during the hospital period (48 hours after the patient's condition was stable)/$\sum$the total number of patients who were hospitalized during the same period)*100%

1. Rate of offerring quit smoking counseling or health benefits education

Rate of offerring quit smoking counseling or health benefits education= ($\sum$patients who received smoking cessation counseling or health benefits education during hospitalization/$\sum$the total number of patients with cerebral infarction who were hospitalized during the same period.)*100%

1. The rate of antihypertensive therapy for cerebral infarction patients with hypertension at discharge

The rate of antihypertensive therapy for cerebral infarction patients with hypertension at discharge= ($\sum$ patients with cerebral infarction complicated with hypertension who were given antihypertensive drugs at discharge /$\sum$the total number of patients with cerebral infarction complicated with hypertension who were hospitalized in the same period)*100%

1. Rate of Patients with diabetes were given hypoglycemic treatment at discharge

Rate of Patients with diabetes were given hypoglycemic treatment at discharge= ($\sum$patients with cerebral infarction complicated with diabetes who were given hypoglycemic drugs at discharge/$\sum$the total number of patients with cerebral infarction complicated with diabetes treated in hospital in the same period)*100%

1. Recurrence rate

Recurrence rate= ($\sum$patients who had recurrent ischemic stroke within 12 month after the first events /$\sum$the total number of patients who were hospitalized during the same period )*100%
