# Supplementary material for: Rationale and design of individualized quality improvement based on the Computer Analysing system to improve Stroke management quality Evaluation (CASE): a multicenter historically controlled study
Source: Trials. 2020 Jul 24;21:677. doi: 10.1186/s13063-020-04598-3 (PMC7379356; doi:10.1186/s13063-020-04598-3)
Supplement: Supplementary file 3 — Additional file 3. The baseline characteristics of primary stroke center (PSC) and comprehensive stroke center (CSC). [file 13063_2020_4598_MOESM3_ESM.docx]

Additional file 3. The baseline characteristics of primary stroke center (PSC) and comprehensive stroke center (CSC)

|  | Entire  N=30 | CSC  N=8 | PSC  N=22 |
| --- | --- | --- | --- |
| Capacity of hospital (beds) |  |  |  |
| Capacity of hospital department (median, beds) | 1343 | 2240 | 1017 |
| Capacity of neurology department (median,beds) | 82 | 117 | 68 |
| Staff of stroke care |  |  |  |
| Stroke team 24*7 on call | 30 (100%) | 8 (100%) | 22 (100%) |
| Neurosurgery 24*7 on call | 30 (100%) | 8 (100%) | 22 (100%) |
| Neurointerventionist on call | 25 (83.3%) | 8 (100%) | 17 (77.3%) |
| Separate neuroradiologist on call | 24 (80%) | 8 (100%) | 16 (72.3%) |
| Professional rehab-physicians or technicians | 29 (96.7%) | 8 (100%) | 21 (95.5%) |
| Team of stroke care |  |  |  |
| Brain CT scan (24*7) | 30 (100%) | 8 (100%) | 22 (100%) |
| CT angiography and interpretation | 25 (83.3%) | 8 (100%) | 17 (77.3%) |
| Transesophageal echocardiography | 22 (73.3%) | 8 (100%) | 14 (63.6%) |
| Complete blood counts test (24*7) | 30 (100%) | 8 (100%) | 22 (100%) |
| Blood biochemistry test (24*7) | 30 (100%) | 8 (100%) | 22 (100%) |
| Rapid blood coagulation test (24*7) | 30 (100%) | 8 (100%) | 22 (100%) |
| Electrocardiogram (24*7) | 30 (100%) | 8 (100%) | 22 (100%) |
| Chest X-ray scan | 30 (100%) | 8 (100%) | 22 (100%) |
| Pathway/procedures of stroke care |  |  |  |
| Intravenous tPA/urokinase thrombolysis | 30 (100%) | 8 (100%) | 22 (100%) |
| Arterial thrombolysis/endovascular therapy | 24 (80%) | 8 (100%) | 16 (72.3%) |
| Carotid artery endarterectomy | 19 (63.3%) | 8 (100%) | 11 (50%) |
| Decompressive craniectomy | 30 (100%) | 8 (100%) | 22 (100%) |
| Stroke unit | 28 (93.3%) | 8 (100%) | 20 (90.1%) |
